# Inflammation-Mediated Genetic and Epigenetic Alterations Drive Cancer Development in the Neighboring Epithelium upon Stromal Abrogation of TGF-β Signaling

**DOI:** 10.1371/journal.pgen.1003251

**Published:** 2013-02-07

**Authors:** B. R. Achyut, David A. Bader, Ana I. Robles, Darawalee Wangsa, Curtis C. Harris, Thomas Ried, Li Yang

**Affiliations:** 1 Laboratory of Cancer Biology and Genetics, Center for Cancer Research, National Cancer Institute, National Institutes of Health, Bethesda, Maryland, United States of America; 2 Department of Molecular and Cellular Biology, Baylor College of Medicine, Houston, Texas, United States of America; 3 Laboratory of Human Carcinogenesis, Center for Cancer Research, National Cancer Institute, National Institutes of Health, Bethesda, Maryland, United States of America; 4 Genetics Branch, Center for Cancer Research, National Cancer Institute, National Institutes of Health, Bethesda, Maryland, United States of America; Centre for Cancer Biology, SA Pathology, Australia

## Abstract

Deletion of tumor suppressor genes in stromal fibroblasts induces epithelial cancer development, suggesting an important role of stroma in epithelial homoeostasis. However, the underlying mechanisms remain to be elucidated. Here we report that deletion of the gene encoding TGFβ receptor 2 (*Tgfbr2*) in the stromal fibroblasts (Tgfbr2^fspKO^) induces inflammation and significant DNA damage in the neighboring epithelia of the forestomach. This results in loss or down-regulation of cyclin-dependent kinase inhibitors p15, p16, and p21, which contribute to the development of invasive squamous cell carcinoma (SCC). Anti-inflammation treatment restored p21 expression, delayed tumorigenesis, and increased survival of Tgfbr2^fspKO^ mice. Our data demonstrate for the first time that inflammation is a critical player in the epigenetic silencing of p21 in tumor progression. Examination of human esophageal SCC showed a down-regulation of TGFβ receptor 2 (TβRII) in the stromal fibroblasts, as well as increased inflammation, DNA damage, and loss or decreased p15/p16 expression. Our study suggests anti-inflammation may be a new therapeutic option in treating human SCCs with down-regulation of TβRII in the stroma.

## Author Summary

Cancer is no longer regarded as a problem of solely cancer cells. The development and metastasis of cancers clearly involves many aspects of the host. We sought to identify the molecular mechanisms underlying epithelial cancer development due to alterations in stromal cells. Using an animal model in which TGF-β signaling is deleted in stromal fibroblasts, we found that inflammation and DNA damage are induced in the epithelial compartment and are responsible for the loss of cell cycle–dependent kinase inhibitors, leading to the compromise of epithelial cell cycle control. These results are important in understanding the stromal-tumor cross talk which has been an important focus in cancer biology in recent years. Our findings suggest that careful examination of the stromal compartment is important and that anti-inflammation therapy may be a new chemoprevention option for epithelial cancer development.

## Introduction

Mounting evidence supports a cross talk between epithelial and stromal cells in cancer progression mediated by paracrine signals and extracellular matrix components [1], [2]. For example, in a prostate cancer model, epithelial tumor progression induces loss of p53 function in stromal fibroblasts [3]. Conversely, in a breast tumor model, deletion of *Pten* in stromal fibroblasts promoted tumor progression associated with massive extracellular matrix (ECM) remodeling, immune cell infiltration, increased angiogenesis, and increased recruitment of the Ets2 transcription factor to targeted gene regulation [4]. Likewise, conditional expression of a mutant allele of *APC* gene in murine uterine stromal cells resulted in endometrial gland hyperplasia progressing to endometrial carcinoma *in situ* and invasive endometrial adenocarcinoma [5]. Furthermore, deletion of *Notch1* in epidermal keratinocytes has significant impact on the stromal microenvironment, promoting skin carcinogenesis [6]. This crosstalk has been witnessed in human sporadic breast cancers: somatic *TP53* mutations in stroma but not epithelia were associated with regional nodal metastases [7]. These studies suggest that epithelial and stromal cell signaling influence one another and may co-evolve during the course of tumor progression [2].

The autocrine and paracrine actions of transforming growth factor-β (TGF-β) have been well documented in stromal and tumor cell interaction [8], [9]. Deletion of *Tgfbr2* in a variety of epithelial cells results in a more aggressive tumor phenotype in mammary, pancreatic, colon, intestinal, head and neck, anal and genital tumors (reviewed by Yang [9]). The mechanism underlying this observation involves increased infiltration of immune cells in the tumor microenvironment [8]–[11]. Interestingly, conditional knockout of the *Tgfbr2* gene in a subset of stromal fibroblasts (FSP1+ cells) contributes to the transformation of epithelia and results in invasive squamous cell carcinoma (SCC) in mouse forestomach [1]. The deletion of *Smad4*, a downstream mediator of TGF-β signaling in T cells, results in spontaneous epithelial cancers throughout the gastrointestinal tract in mice [12]. Unexpectedly, epithelial-specific deletion of *Smad4* did not result in the same tumor phenotype [12]. The impact of stromal TGF-β on epithelial cancers was also demonstrated in a tissue recombination model wherein loss of TβRII function in 50% of immortalized human prostate fibroblasts resulted in malignant transformation of the nontumorigenic human prostate epithelial cells [13]. These studies suggest that stromal loss of TGF-β signaling induces epithelial transformation. One of the mechanisms delineated in these studies involves hepatocyte growth factor (HGF) overproduction by Tgfbr2^fspKO^ stroma and activation of c-MET signaling on adjacent epithelia through paracrine signaling, resulting in epithelial hyperproliferation [1], [14]. However, it is unclear whether changes in stromal cells induce genetic and epigenetic alterations in the epithelial compartment, and if so, what are the underlying molecular mechanisms?

Here we report that stromal deletion of *Tgfbr2* induced inflammation resulted in DNA damage, loss of p15 and p16, promoter methylation of p21, and increased epithelial proliferation, i.e. the development of SCC. We showed for the first time that down-regulation of TGF-β signaling in the stroma has significant impact on the genetic and epigenetic components of the adjacent epithelial compartment through inflammation mediated mechanisms. Therefore, therapeutic targeting of inflammation may be a useful strategy in treating human SCCs with down-regulation of TβRII in the stroma.

## Results

### Deletion of *Tgfbr2* in FSP1+ Stromal Cells Induces Loss of p15 and p16 in the Neighboring Epithelial Compartment

**Figure 1:**
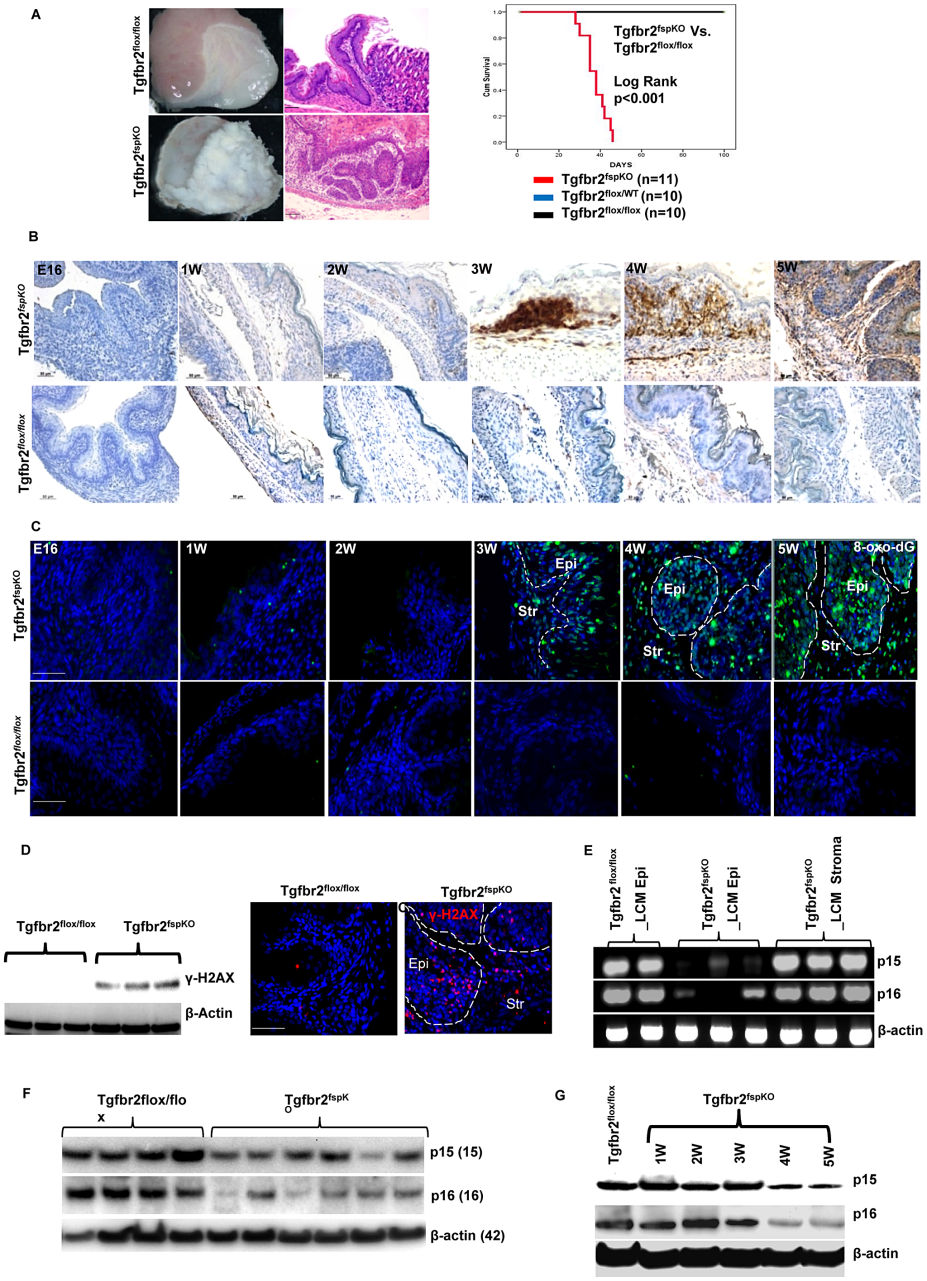
Stromal deletion of Tgfbr2 promotes inflammation-induced DNA damage and loss of *p15 and p16*. (A) Tgfbr2^fspKO^ mice developed forestomach SCC with 100% penetrance (left panel, scale bar: 50 µm). Mice died within 7 weeks with a median survival of 38 days (Kaplan Meier survival curve, Log rank p<0.001) (right panel). (B) IHC of CD45+ leukocytes in forestomach tissues of Tgfbr2^fspKO^ and Tgfbr2^flox/flox^ mice from embryonic day 16 (E16) to 5 weeks. Scale bar: 50 µm. (C) Immunofluorescence staining of 8-oxo-dG of forestomach samples of Tgfbr2^fspKO^ and Tgfbr2^flox/flox^ mice from E16 to 5 weeks. Scale bar: 100 µm. (D) Western blot of γ-H2AX from protein samples of Tgfbr2^fspKO^ and Tgfbr2^flox/flox^ forestomach (n = 3 mice for each group) (left panel), with Immunofluorescence staining of γ-H2AX on the right panels. Scale bar: 100 µm. (E) Genomic PCR of p15 and p16 from epithelial and stromal cells laser-dissected from Tgfbr2^fspKO^ and Tgfbr2^flox/flox^ forestomach tissues. The input for genomic PCRs was normalized with β-actin. (F) Western blot of p15 and p16 in the epithelial layer of forestomach from Tgfbr2^fspKO^ (n = 6) and Tgfbr2^flox/flox^ mice (n = 4). (G) Western blot of time course studies of p15/p16 expression in the epithelial layer of forestomach, showing reduced expression of p15 and p16 from 4 week of age in the Tgfbr2^fspKO^ mice. Str: Stroma; Epi: epithelia.

Stromal cells and their signaling pathways have significant impact on epithelial tumor progression [4], [12], [13]. Specific deletion of *Tgfbr2* in FSP1+ fibroblasts (Tgfbr2^fspKO^) induced development of SCC in forestomach with 100% penetrance [1] (Figure 1A, left panel). These mice die by 7 weeks with a median survival of 38 days (Log rank p<0.001) (Figure 1A, right panel). Examination of Tgfbr2^fspKO^ forestomach between embryonic day 16 (E16) and 5 weeks of age suggested that hyperplasia began during week 3 and was followed by dysplasia, carcinoma *in situ*, and invasive SCC (Figure S1A). Here we investigated the molecular mechanisms that are responsible for the development of SCC due to loss of *Tgfbr2* in the stromal compartment. We first confirmed the specific deletion of *Tgfbr2* in stromal fibroblasts using TβRII immunofluorescence (Figure S1B) and β-galactosidase IHC in FSP1-Cre/Rosa26 reporter mouse tissue (Figure S1C). The absence of p-smad2 nuclear localization in stroma was used as an indicator for the absence of TGF-β signaling (Figure S1D) [1].

Tgfbr2^fspKO^ SCC tumors showed substantial infiltration of CD45+ leukocytes between weeks 3 and 5 compared to Tgfbr2^flox/flox^ littermates (Figure 1B), indicating an inflammatory reaction due to loss of *Tgfbr2* in stromal fibroblasts. Inflammation is a critical player in carcinogenesis and is known to cause DNA damage as well as histone modification in cancer [15], [16]. We thus examined DNA damage in forestomach sections of Tgfbr2^fspKO^ and Tgfbr2^flox/flox^ mice using immunofluorescence staining of 8-oxo-2′-deoxyguanosine (8-oxo-dG), a major product of DNA oxidation indicative of DNA damage. Interestingly, DNA damage was initially detected in mice at 3 weeks of age and became progressively worse by 5 weeks (Figure 1C) concomitant with infiltration of CD45+ leukocytes. The expression of γ-H2AX (Figure 1D), a histone molecule associated with DNA double strand breaks, was also increased in Tgfbr2^fspKO^ mice. The 8-oxo-dG and γ-H2AX were not seen in the forestomach of Tgfbr2^flox/flox^ control mice (Figure 1D, left and middle panels). Our data suggest that loss of *Tgfbr2* in FSP1+ stromal cells induced inflammation and DNA damage.

DNA damage often results in chromosomal aneuploidy [17] and alteration of epigenetic marks including acetylation, methylation, and ubiquitylation [18]. We evaluated genetic alterations using array-CGH and genomic DNA PCR. We first analyzed epithelial cells isolated from forestomach tumors of Tgfbr2^fspKO^ mice. We found a loss of band C4 of chromosome 4 (Figure S2A), which contains CDK inhibitors *Cdkn2b/p15^INK4B^* (p15), *Cdkn2a/p16^Ink4A^* (p16), and *Cdkn2a/p19Arf*. Loss of p15 and p16 tumor suppressor genes is a frequent event in human and mouse cancers [19], [20]. We confirmed loss of p15 and p16 using array-CGH and genomic PCR of epithelial cells from tumor tissue sections of 5 week old Tgfbr2^fspKO^ mice using laser capture microdissection technology (Figure S2B and S2C, Figure 1E). *Cdkn2a/p19^Arf^* is an alternative reading frame in the same locus that harbors p16, it is presumably deleted along with p16. These genetic alterations were not detected in the stromal compartment of the forestomach sections from Tgfbr2^fspKO^ mice (Figure S2C and Figure 1E). Western analysis of forestomach tumor tissues from Tgfbr2^fspKO^ mice showed loss of or decreased expression of p15 and p16 proteins (Figure 1F), which was detected from 4 week of age (Figure 1G), one week after the inflammation onset (Figure 1B). p15 and p16 are critical mediators in cell cycle control and are important in suppressing tumor development [19]. Our data suggest that deletion of *Tgfbr2* in FSP1+ stromal cells induced a loss of p15 and p16 in epithelial cells.

### Alteration of Cell Cycle Mediators and Increased Proliferation in the Forestomach Epithelia of Tgfbr2^fspKO^ Mice

**Figure 2:**
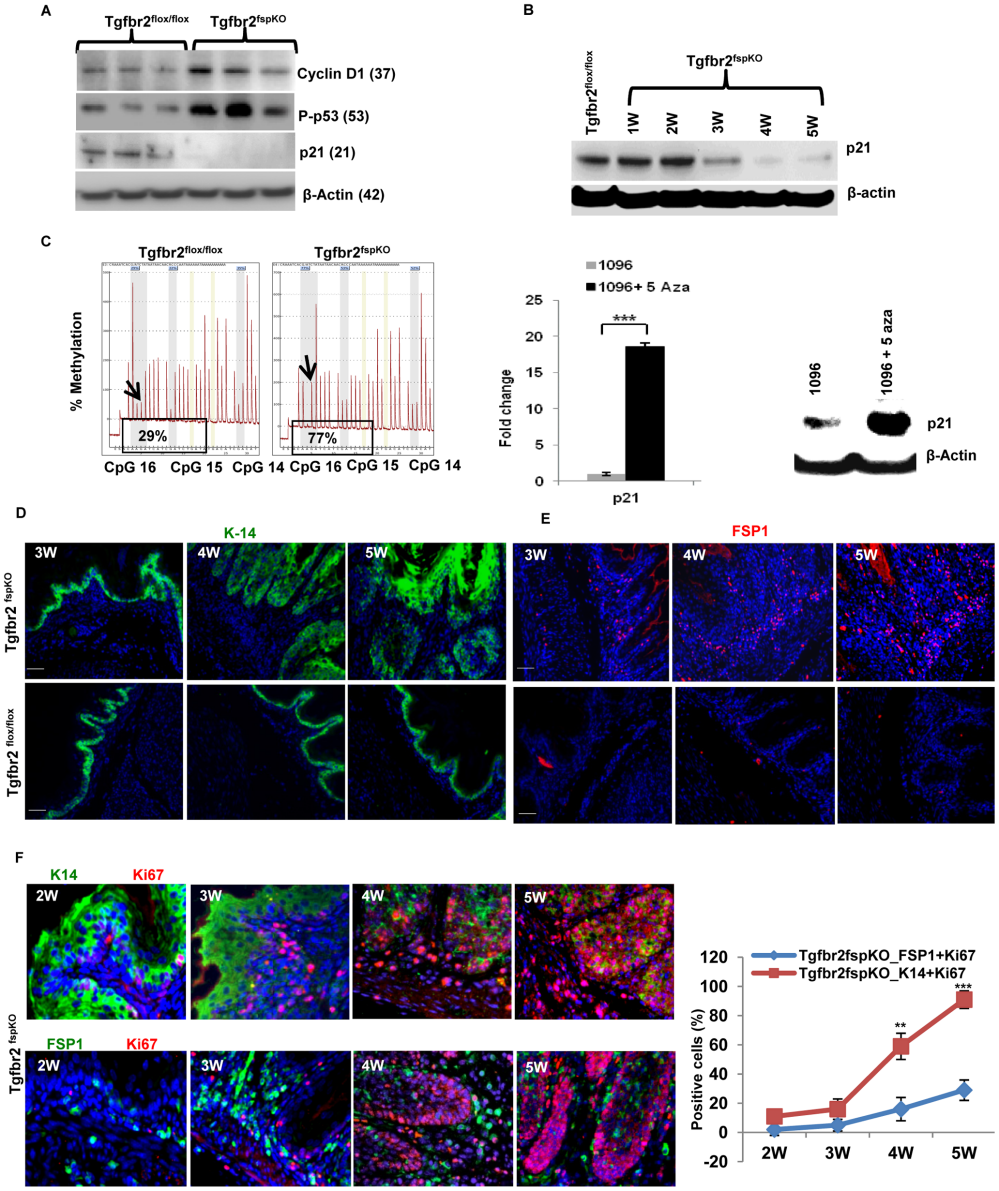
Methylation of *p21* promoter, cell cycle alterations, proliferation, and SCC development. (A) Western blot of cyclin D1, p53 and p21. Proteins were extracted from the epithelial layer of forestomach tissue of Tgfbr2^fspKO^ and Tgfbr2^flox/flox^ mice (n = 3 for each group). (B) Time course studies of p21 expression by Western blot, showing p21 expression reduced in the forestomach of Tgfbr2^fspKO^ mice at 3 week of age but with more profound reduction at 4 weeks. (C) Pyrosequencing data showing % methylation of p21 promoter (CpG 16) in forestomach tumor samples of *Tgfbr2^fspKO^* (77%) compared to that of *Tgfbr2^flox/flox^* mice (29%), left panel; increased expression of p21 mRNA (Q-PCR, middle panel) and protein (Western blot, right panel) after 5-aza 2′ deoxycytine treatment. Epithelial cells 1096 was isolated from Tgfbr2^fspKO^ mice and treated at 5 µM dose for 48 hours. (D and E) Immunofluorescence staining of K-14 (D, green) and FSP1 (E, red) in forestomach tissues of Tgfbr2^fspKO^ and Tgfbr2^flox/flox^ mice at 3, 4, and 5 weeks. Scale bar: 50 µm. (F) Co-immunofluorescence staining of Ki-67 (red) with K-14 or FSP-1 (green) showing proliferation in epithelial and stromal compartments in Tgfbr2^fspKO^ mice. Scale bar: 150 µm. **P<0.01 and ***P<0.001. Three mice were evaluated. Shown is one of the two experiments performed.

Our data suggest a dysregulation of the G_1_ cell cycle checkpoint in epithelial cells due to loss of *Tgfbr2* in FSP1+ stromal cells. We next examined several critical molecules in cell cycle control. The expression of Cyclin D1 was increased in the forestomach of 5 week old Tgfbr2^fspKO^ compared to Tgfbr2^flox/flox^ mice (Figure 2A). Likewise, expression of phospho-p53 (p-p53) was increased (Figure 2A), likely in response to DNA damage (Figure 1C). No mutation was found in p53 (data not shown). Surprisingly, expression of *Cdkn1a/p21* (p21), the downstream mediator of p53, was reduced in the forestomach of Tgfbr2^fspKO^ mice at 3 weeks of age but with more profound reduction at 4 weeks (Figure 2A and 2B). Suspecting epigenetic regulation of p21 expression in tumor cells, we performed pyrosequencing and found an increased methylation of CpG in the p21 promoter in forestomach tumor samples of *Tgfbr2^fspKO^* (77%) compared to that of *Tgfbr2^flox/flox^* mice (29%) (Figure 2C, left panel). We then treated forestomach tumor epithelial cells (1096, isolated from SCC of FSP-Cre/RII knock out mouse, and kept in low passages) with the DNA methyltransferase inhibitor 5-aza 2′ deoxycytidine (Decitabine) at 5 uM concentration for 48 hours. We observed increased expression of p21 at both the mRNA and protein level, and decreased cell proliferation compared to untreated cells (Figure 2C, right panel, and Figure S3A). Our data suggest that methylation of the p21 promoter likely prevented p53-mediated p21 transcription, resulting in decreased expression of p21 in Tgfbr2^fspKO^ mice. Together, these data support a loss of cell cycle mediators in epithelial cells due to a loss of *Tgfbr2* in stromal compartment in Tgfbr2^fspKO^ mice.

We next evaluated cell proliferation in the forestomach of Tgfbr2^fspKO^ and control mice as downregulation of p15, p16 and p21 may lead to increased proliferation. Forestomach tissue from 3, 4, and 5-week old mice, was collected and stained with FSP1 (stroma) and cytokeratin-14 (K14) (epithelium). Using immunofluorescence, we observed hyperplasia at 4 weeks and dysplasia/carcinoma *in situ* at 5 weeks in the epithelial compartment (Figure 2D), and increased FSP1+ cells in Tgfbr2^fspKO^ mice (Figure 2E). The latter was likely due to deletion of *Tgfbr2* and loss of growth inhibition. Co-staining of Ki-67, a marker of cellular proliferation, with K14 or FSP1 showed the extent of proliferation was more pronounced in the epithelial compartment compared to the stromal compartment (Figure 2F). Interestingly, the epithelial compartment had very few apoptotic cells compared to the stromal compartment (Figure S3B). Together, these data suggest that absence of TGF-β signaling in stromal fibroblasts likely induced uncontrolled proliferation and decreased apoptosis in the epithelial compartment.

### Inflammatory Mediators and the Tumor Microenvironment of SCC in Tgfbr2^fspKO^ Mice

**Figure 3:**
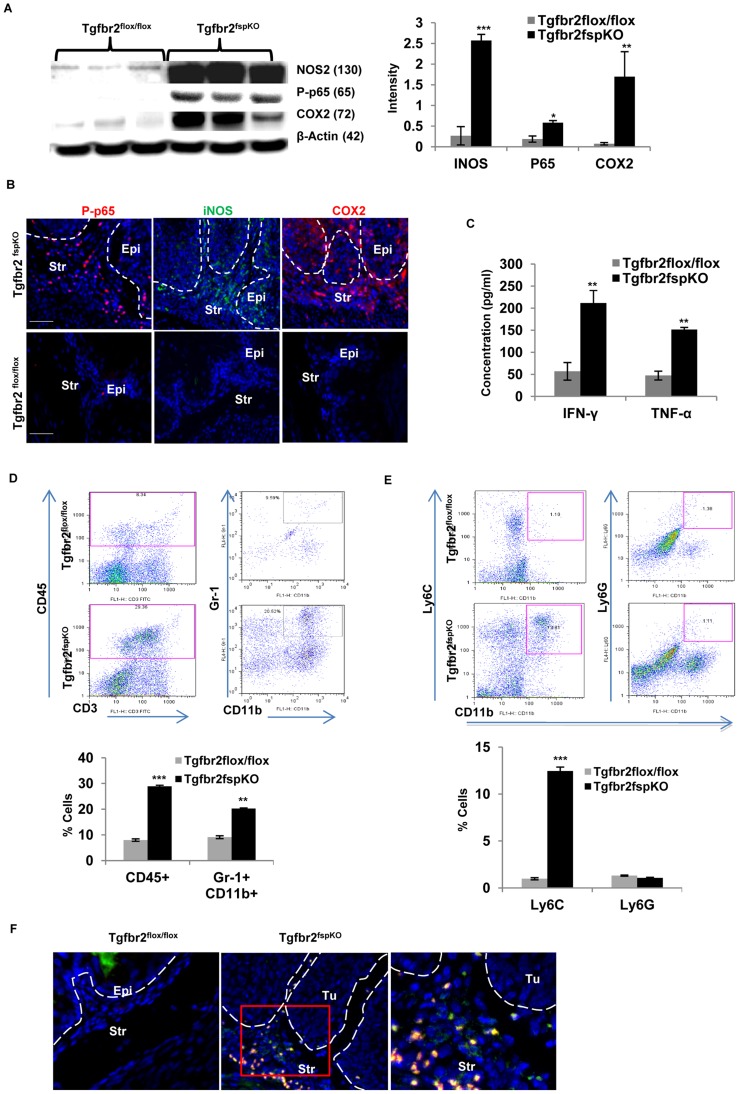
Inflammatory mediators and tumor microenvironment in SCC. (A) Western analysis of iNOS, COX2, and p65 in tumor tissue and equivalent normal tissue layer of 5 week old Tgfbr2^fspKO^ and Tgfbr2^flox/flox^ mice (n = 3 for each group), semi-quantitative data from band density is on the right panel (right panel). (B) Immunofluorescence staining of iNOS, COX2, and p65 of forestomach in 4-week-old Tgfbr2^fspKO^ mice compared to Tgfbr2^flox/flox^ control mice. (C) Bioplex assay of IFN-γ and TNF-α of forestomach samples of 5 week old Tgfbr2^fspKO^ and Tgfbr2^flox/flox^ mice. (D) Flow cytometry of CD45+ and Gr-1+CD11b+ cells from single cell suspension of forestomach of Tgfbr2^fspKO^ and Tgfbr2^flox/flox^ mice (dot plots in left panels), with quantitative data in the lower panel. (E) Flow cytometry analysis of LY6C+ and LY6G+ subsets of Gr-1+CD11b+ cells from (D), quantitative data is in the lower panel. (F) Immunofluorescence staining of CD4 and IL17A of forestomach in 5-week-old Tgfbr2^fspKO^ mice compared to Tgfbr2^flox/flox^ control mice. Error bars represent standard deviation (SD). *P<0.05, **P<0.01 and ***P<0.001. Three mice were evaluated in each group. Shown is one of the two experiments performed. Str: Stroma; Epi: epithelia.

Inflammation has been shown to play an important role in carcinogenesis. To characterize the role of inflammation in Tgfbr2^fspKO^ mice, we collected forestomach tissue from 4 week old Tgfbr2^fspKO^ and Tgfbr2^flox/flox^ control mice for Western analysis. We found an increased expression of inducible nitrogen oxide synthatase (NOS2), cyclooxygenase 2 (COX2), and nuclear factor (NF) κB subunit (p65) in forestomach epithelail layer samples of 4 week old Tgfbr2^fspKO^ mice compared to control mice (Figure 3A, left and right panels). The increased expression of NOS2, COX2, and p65 was found in both the stromal and epithelial compartments (Figure 3B). IFN-γ and TNF-α were significantly increased in the tumor tissues (Figure 3C), suggesting a type I inflammatory response associated with the development of SCC frequently observed in gastrointestinal cancers [21].

Inflammation promotes migration and infiltration of leukocytes, certain types of which are known to have significant impact on tumor microenvironment and tumor progression. We further characterized the subsets of infiltrating immune cells described earlier (see figure 1). We found a significant increase in CD45+ cells in the forestomach of 5-week-old Tgfbr2^fspKO^ compared to control Tgfbr2^flox/flox^ mice (29.36% vs 8.34%) (Figure 3D). The increased immune cells included Gr-1+ CD11b+ cells (20.52% vs 9.59%), also known as myeloid derived suppressor cells (MDSCs). These MDSCs are mostly monocytic subset Ly6C+CD11b+ (13.81% vs 1.10%) but not Ly6G+CD11b+ cells (Figure 3E). MDSCs have significant impact on tumor microenvironment [8], [21], [22] and are well known for their role in cancer associated immune suppression [23]. In addition, there was a significant presence of TH17 cells that stained for CD4+IL17A+ by immunofluorescence staining in 5-week-old Tgfbr2^fspKO^ compared to control Tgfbr2^flox/flox^ mice (Figure 3F). These findings reveal considerable alteration in the cellular and molecular properties of the tumor microenvironment in Tgfbr2^fspKO^ compared to Tgfbr2^flox/flox^ mice, suggesting that multiple inflammatory mediators interacting with an altered microenvironment are implicated in the progression of SCC following deletion of TGF-β signaling in stromal fibroblasts.

### Anti-Inflammation Treatment Significantly Delayed SCC Development and Prolonged Survival of Tgfbr2^fspKO^ Mice

**Figure 4:**
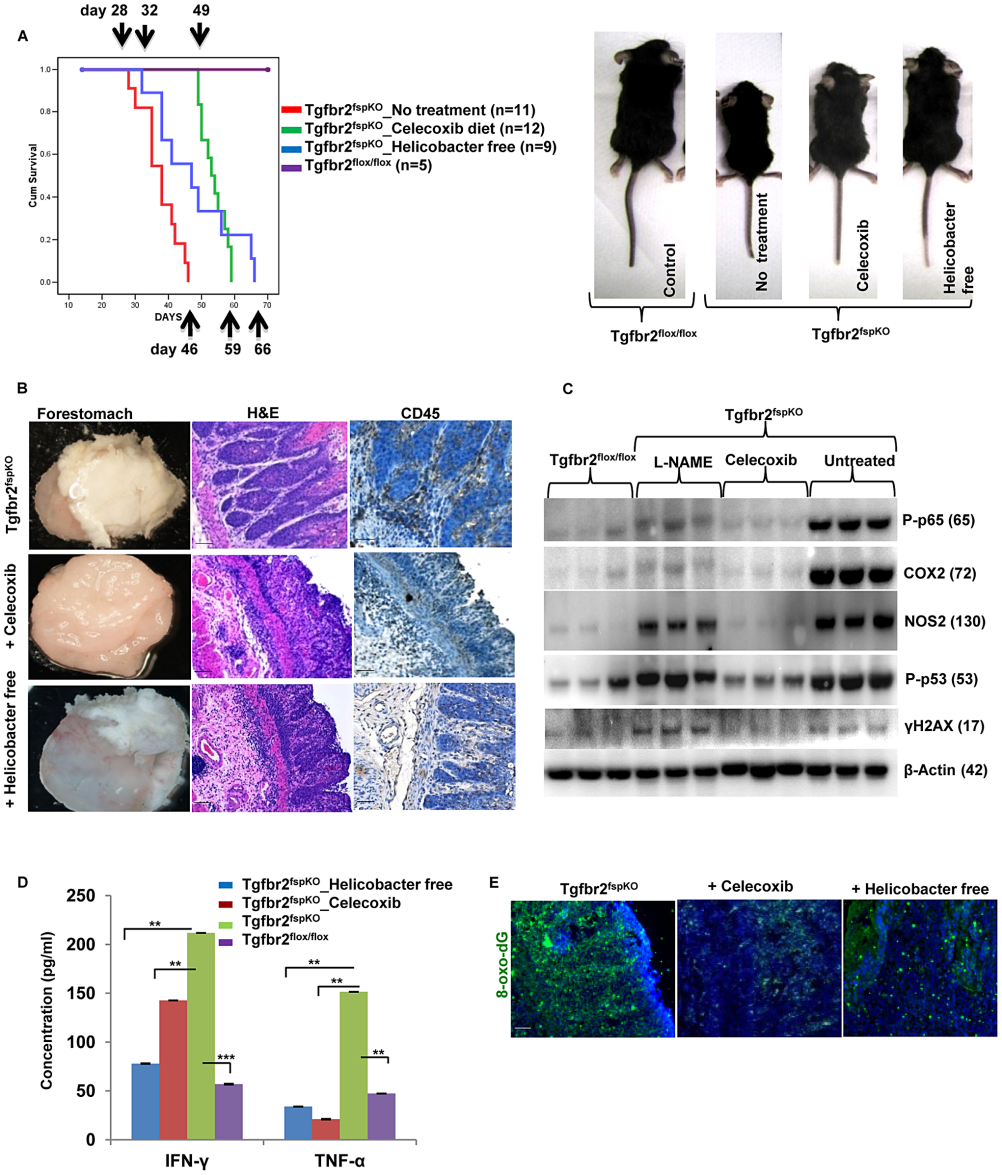
Anti-inflammation delays SCC development and prolongs survival of Tgfbr2^fspKO^ mice. (A) Kaplan survival curve of Tgfbr2^fspKO^ mice treated with Celecoxib or housed in Helicobacter free environment. The pups received the Celecoxib starting the second week after birth, together with the nursing mother mouse in the same cage. The treatment continued after weaning. (B) Celecoxib-treated and Helicobacter free Tgfbr2^fspKO^ mice displayed decreased hyperplasia and CD45+ infiltration by histopathology compared to untreated Tgfbr2^fspKO^ mice. Forestomach samples from 5 week mice stained for H&E and CD45. Scale bar: 20 µm. (C) Western blot analysis of iNOS, COX2, p65, γ-H2AX and p53 in forestomach samples from Tgfbr2^fspKO^ mice treated with Celecoxib or L-NAME. (D) IFN-γ and TNF-Bioplex assay with forestomach samples from Tgfbr2^fspKO^ and Tgfbr2^fspKO^ mice treated with Celecoxib or housed in Helicobacter free condition. Error bars represent SD. **P<0.01 and ***P<0.001. (E) Reduced production of 8-oxo-dG in Tgfbr2^fspKO^ mice treated with Celecoxib or in Helicobacter free conditions. Scale bar: 20 µm.

To investigate the role of inflammation in SCC development, Tgfbr2^fspKO^ mice were treated with the COX2 inhibitor (Celecoxib). Celecoxib treatment significantly improved body size and weight, decreased tumor burden, and increased lifespan of Tgfbr2^fspKO^ mice from 28 to 49 days (Figure 4A, left and right panels and Figure S4A and S4B). The median survival of Celecoxib treated Tgfbr2^fspKO^ mice was 53 days compared to 38 days in untreated mice (Log rank p<0.001) (Figure 4A, left and right panels and Figure S4A and S4B). This was accompanied by reduced infiltration of CD45+ leukocytes and decreased hyperplasia (Figure 4B). Surprisingly, treatment with L-NAME, an inhibitor of NOS2, did not significantly affect body weight and tumor burden or lifespan despite decreased serum levels of nitric oxide to normal baseline (Figure S4A and S4B, Figure S5A and S5B).

Alterations in microbial communities, particularly in gastrointestinal (GI) tract, are associated with inflammation and cancer development [24]. To investigate the role of microbiome in the progression of SCC in the Tgfbr2^fspKO^ model, we re-derived the mouse line using super-ovulation and artificial insemination to obtain pups free of Helicobacter (Table S1). The uninfected Tgfbr2^fspKO^ mice displayed significantly improved body size and weight with a median survival time of 47 days compared to 38 days in mice housed under standard conditions (Log rank p<0.001) (Figure 4A, left and right panels and Figure 4B). The tumors in the uninfected mice were characterized by decreased CD45 infiltration, decreased production of COX2 and P-p65 (Figure 4B and Figure S5C); delayed hyperplasia and dysplasia compared to the control mice (Figure 4B). These data suggest an involvement of microflora in the inflammation and SCC development in Tgfbr2^fspKO^ mice. Indeed, alterations in the microflora are associated with inflammation and intestinal metaplasia of the distal esophagus [25].

In addition to a decrease in p53 expression (Figure 4C), Celecoxib treatment decreased COX-2, NOS2 and p65 expression to levels similar to control samples (Figure 4C and Figure S5C). L-NAME treatment decreased the expression of COX-2, NOS2 and p65. However, it did not affect p53 and γ-H2AX production (Figure 4C). Celecoxib treatment and Helicobacter free environment significantly reduced IFN-γ and TNF-α levels (Figure 4D), and decreased 8-oxo-dG production in the forestomach of Tgfbr2^fspKO^ mice (Figure 4E). These data suggest that COX-2 and Helicobacter infection are important mediators in inflammation and SCC progression.

### Celecoxib Treatment Significantly Restored the Expression p21 in Tgfbr2^fspKO^ Mice

**Figure 5:**
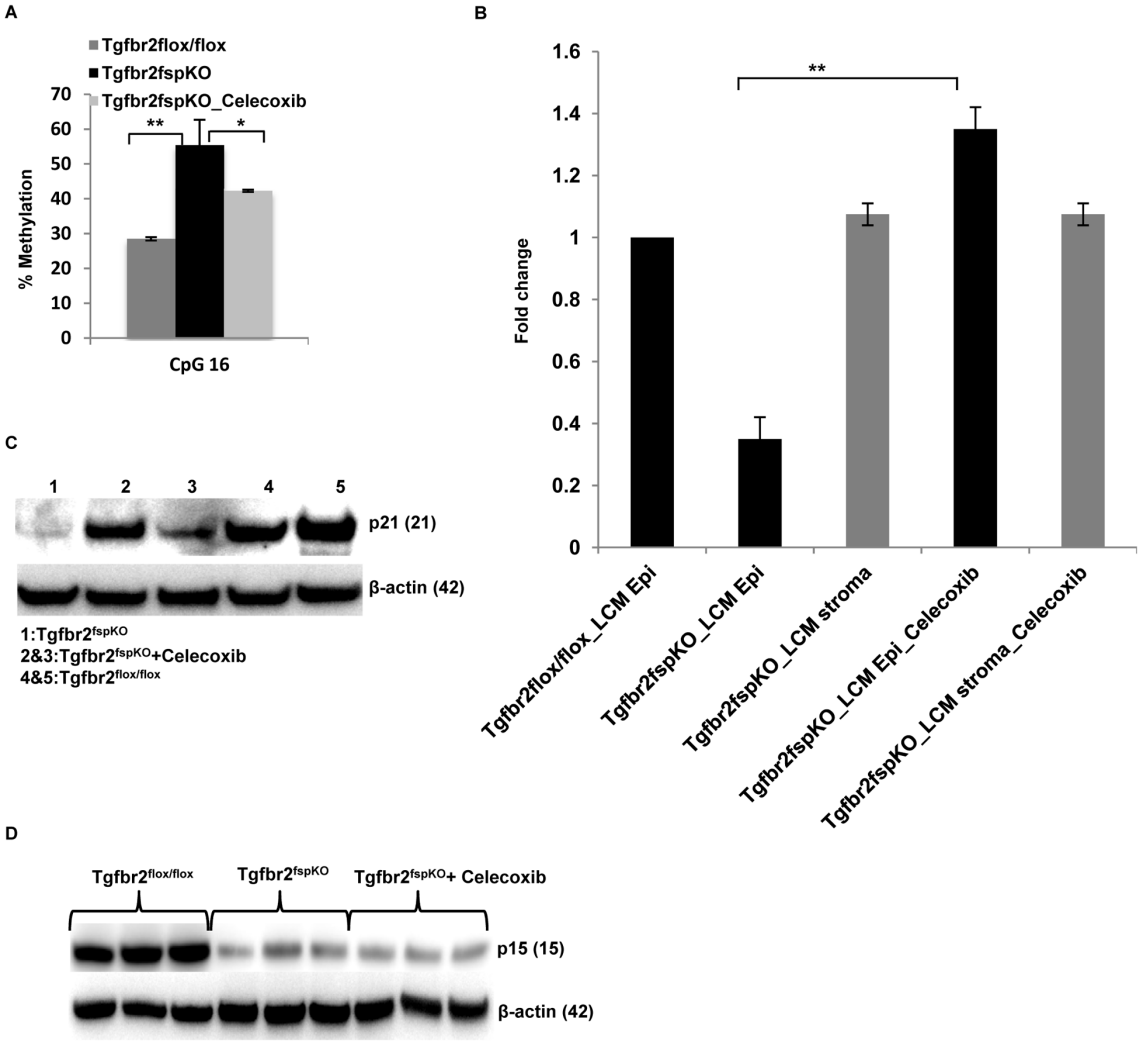
Anti-inflammation decreases promoter methylation and restores *p21* expression. (A) Decreased CpG 16 methylation of p21 promoter from forestomach samples of *Tgfbr2^fspKO^* mice treated with Celecoxib (n = 3). Data is represented as mean ± SD. (B) Real time RT-PCR of p21 expression in laser captured epithelial and stromal cells from Tgfbr2^flox/flox^ and Tgfbr2^fspKO^ mice, treated with Celecoxib. (C) Western blot of p21 in forestomach samples of Tgfbr2^fspKO^ mice treated with Celecoxib (n = 2) compared to untreated Tgfbr2^fspKO^ mice (n = 1) and floxed control mice (n = 2). (D) Celecoxib treatment of Tgfbr2^fspKO^ mice did not restore the expression of p15 in epithelial layers of forestomach tumors (Western blot). *P<0.05 and **P<0.01.

We showed earlier that the downregulation of p21 was likely mediated by methylation of p21 promoter. We next investigated whether anti-inflammatory treatment would decrease this methylation and increase p21 expression. Celecoxib treated Tgfbr2^fspKO^ mice were evaluated for p21 promoter methylation by pyrosequencing of the epithelial layer from the forestomach tissue. Interestingly, methylation was significantly decreased (p<0.05) at CpG 16 (Figure 5A). Consistent with this finding, the p21 mRNA expression was observed in the laser captured epithelia from Celecoxib treated Tgfbr2^fspKO^ mice (Figure 5B). The p21 mRNA in the stroma was not different from that of the Tgfbr2^flox/flox^ control mice, and was not changed by Celecoxib treatment (Figure 5B). p21 protein expression was also increased upon Celecoxib treatment (Figure 5C). In contrast, p15 expression was not observed (Figure 5D). Together with genomic PCR showing a loss of p15 (Figure 1E), our data indicate that p15 might be genetically deleted. Surprisingly, Celecoxib treatment also resulted in increased expression of p16 protein (data not shown), suggesting a possible methylation of p16 promoter as a result of inflammation, similar to that of p21. Together, our data support that inflammation induced DNA damage, genetic and epigenetic alterations of cell cycle mediators play a critical role in SCC progression in Tgfbr2^fspKO^ mice.

### Human Esophageal Squamous Cell Carcinoma (ESCC) Exhibited a Decreased Expression of TβRII in FSP1+ Stromal Cells, Increased Inflammation, and Elevated Production of 8-oxo-dG

**Figure 6:**
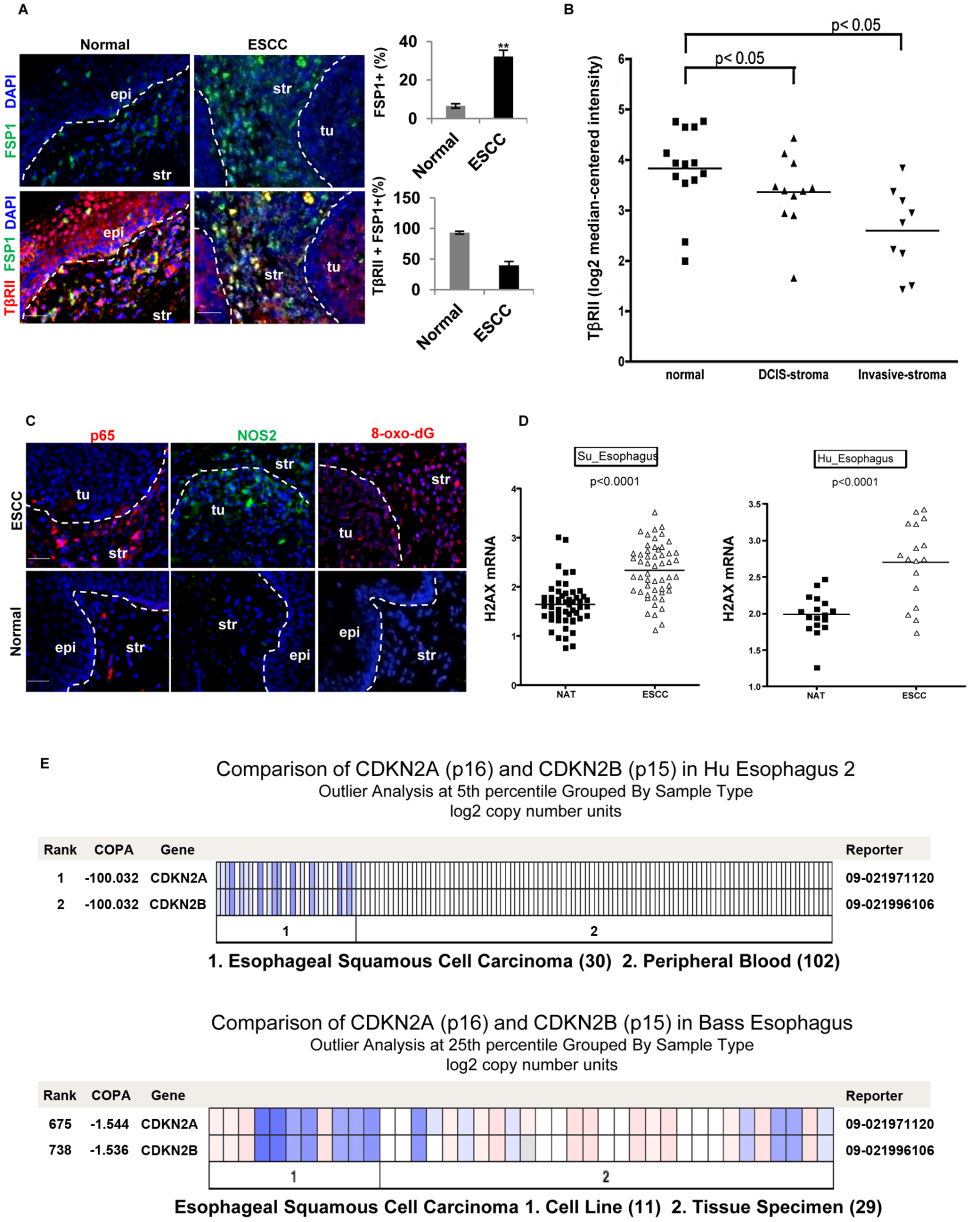
Reduced expression of TβRII in FSP1+ stromal cells, evident inflammation and DNA damage in human ESCC. (A) Immunofluorescence staining of FSP1+ cells (green) and TβRII (red) in human ESCC and adjacent normal esophagus (left panels). Quantitative data are listed on the right panels. The % FSP-1+ is the FSP1+ cells out of all the cells in one field using Image J software. The % FSP-1+TBRII+ cells are the double positive cells in all FSP+ single positive cells. Three different fields were evaluated, and the average was obtained and shown. Scale bar: 20 µm. Error bars represent SD. **P<0.01. (B) Down-regulation of TβRII in tumor-associated stroma compared to the adjacent normal in a dataset of breast carcinoma. Log2 median-centered intensity RNA expression values were downloaded from Oncomine mRNA dataset Ma 4 Breast, used to generate a dot plot in GraphPad Prism 5.0 and analyzed by two-tailed, paired t-test (right panel). (C) Immunofluorescence staining of p65, iNOS, and 8-oxo-dG in human ESCC. Representative images are shown. Scale bar: 50 µm. Str: Stroma; Epi: epithelia. (D) Significant up-regulation of γ-H2AX mRNA in ESCC compared to normal adjacent tissue (NAT). Data from two ESCC published studies that feature paired ESCC and NAT mRNA microarray were analyzed (www.oncomine.com). (E) Heat map showing copy number loss of p15 and p16 in ESCC. This data was obtained from the same dataset as in (D) which measure DNA copy number on a SNP microarray platform. Blue depicts copy number loss, compared to no loss (white) in peripheral blood DNA.

The SCC in the forestomach of the Tgfbr2^fspKO^ mice shows similarity to that of human ESCC by way of similar histology and functional behavior. Additionally, downregulation of TGF-β receptors has previously been reported at the invasive front and stroma in human ESCC [26] and prostate cancer [13], [27]. Due to these histological and molecular similarities, we measured the TβRII expression level in FSP1+ stromal cells of eight human ESCC specimens. Adjacent normal tissues from these patients served as a control (Figure S6A). We observed an increased number of FSP1+ cells in the stromal compartment of the ESCC tumors compared to the adjacent normal esophagus (32.2% vs 6.6%, p<0.001) (Figure 6A, upper panels). This data was consistent with the expansion of FSP1+ cells in Tgfbr2^fspKO^ mice (Figure 2E). In these FSP1+ cells, TβRII expression was decreased in tumor esophagus compared to adjacent normal esophagus (39.8% vs 93.2%, p<0.001) (Figure 6A, lower panels). Down-regulation of TβRII was also observed in tumor-associated stroma compared to the adjacent normal in a dataset of breast carcinoma (Ma 4 Breast) (Figure 6B). The expression of p65 and NOS2 was elevated, 8-oxo-dG was increased in both stromal and epithelial compartments (Figure 6C), consistent with findings in the animal model. In order to investigate biomarkers of DNA damage and genetic aberrations in human ESCC, we interrogated the Oncomine database (www.oncomine.com). Expression of H2AX mRNA was significantly upregulated in ESCC (p<0.0001) (Figure 6D). Additionally, p15 and p16 were co-deleted in human ESCC (Figure 6E) and [28]–[30]. These data suggest an association of reduced expression of TβRII in stromal cells with increased inflammation, DNA damage, and genetic alterations in human ESCC, which is consistent with our observations in Tgfbr2^fspKO^ mice.

## Discussion

**Figure 7:**
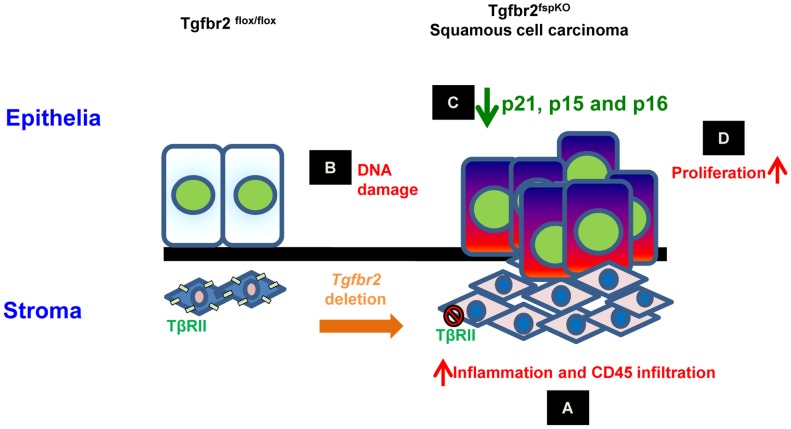
Schematic hypothesis for the development of forestomach SCC due to loss of *Tgfbr2* in stromal fibroblasts. (A) *Tgfbr2* deletion in FSP1+ fibroblasts results in inflammation and infiltration of CD45+ cells in the microenvironment; (B) Inflammation induces DNA damage characterized by 8-oxo-dG and H2AX production in epithelia; (C) epigenetic silencing of p21 and p16, and genetic loss of p15 and p16 in epithelia; (D) loss of cell cycle control and hyper-proliferation of epithelia, development of SCC.

Significant cross-interactions between stroma and tumor cells have been reported in recent studies [1]–[4]. Alteration of tumor suppressor genes in stromal fibroblasts induces epithelial cancer development [4], [5], [7], suggesting an important role of stroma in epithelial homoeostasis. Deletion of TGF-β signaling in the stroma modulates oncogenic potential of neighboring epithelia and induces the development of SCC in the forestomach in a mouse model (Tgfbr2^fspKO^ mice) ^1^. However, the underlying mechanisms are unclear. In this report, we demonstrate that deletion of *Tgfbr2* in the stromal fibroblast resulted in severe inflammation and DNA damage, which induced lost or decreased expression of p15, p16, as well as p21 in the epithelial cell compartment and the subsequent SCC development in forestomach (Figure 7). Our studies provide mechanistic insight to how loss of tumor suppressor in the stromal cells impact epithelial tumorigenesis and progression.

### FSP1-cre Mediated Specific Deletion of *Tgfbr2*

We used FSP1-cre transgenic mice to mediate *Tgfbr2* deletion specifically in the stromal fibroblasts. However, no alteration in epithelial TβRII was noticed. We used several approaches including TβRII immunofluorescence, p-smad2 nuclear localization in stroma, and an FSP1-Cre/Rosa26 reporter mouse to verify specificity. FSP1/S100A4 was identified as a specific marker for fibroblasts [31]. The FSP1-cre mediated gene deletion has been widely used in a number of mouse models for fibroblast specific gene deletion [1], [4], [32]. However, several studies reported that a specific subset of inflammatory macrophages co-express FSP1 and F4/80+ under a number of pathological conditions [33], [34]. We did not observe an overlap between FSP1+ cells with F4/80 positive cells (Figure S6B). This is supported by an extensive study regarding comparison overlap between FSP1+ fibroblasts with the macrophage markers, in which they authors demonstrated that F4/80 antibodies can be used to distinguish macrophages from FSP1+ fibroblasts [35]. We thus believe that the FSP1-cre mediated deletion is highly specific in a subset of stromal fibroblasts, with a small possibility in other host cells, and certainly not in tumor cells.

### Loss of TGF-β Signaling Induced Inflammation and DNA Damage

TGF-β signaling regulates inflammation, as deletion of *Tgfbr2* increased the transcription factor NF-κB [36]. In TGF-β1 deficient mice, inflammation causes precancerous lesions to progress to colon cancer [37]. Inflammation is known to induce DNA damage, genetic alterations and histone posttranslational modifications in mice and human cancers [15], [16], [20], [38]. We found a significant production of 8-Oxo-dG and γ-H2AX, indicating severe DNA damage from 3 weeks of age in the Tgfbr2^fspKO^ mice. This is likely due to production of reactive oxygen and nitrogen species such as nitrogen oxide (NO). DNA damage often results in chromosomal segregation errors and structural alterations including mutations, deletions, amplifications, and balanced/unbalanced chromosomal translocations [17], [20], [39]–[41]. Indeed, we found a loss of p15 and p16 located in mouse chromosome 4 band C4 locus, which is orthologous to chromosome band 9p21 in humans. The loss of these chromosome bands is frequently observed in murine and human cancers [20], [42], [43]. Inflammatory mediated genetic alterations were previously observed in the head and neck tumor mice model, in which SMAD4−/− deletion in head and neck epithelia resulted in genetic aberrations and deletion of chromosome 4q in SMAD−/− mice [20]. Conditional deletion of p120 in the esophagus, oral cavity, and forestomach increased the production of proinflammatory cytokine TNF-α [21]. TNF-α, and IFN-γ, are known to induce epithelial dysfunctions and SCC of intestine [44], [45]. Our data and these published reports may provide molecular insight for a previous study that showed inactivation of *Smad4* and *PTEN* in K5+ epithelia induced forestomach SCC development and downregulation of CDK inhibitors [46].

### Inflammation, Epigenetic Silencing of p21, and Lost Cell Cycle Control

Deletion of *Tgfbr2* induced inflammation was also responsible for the decreased p21 expression in the Tgfbr2^fspKO^ mice. Very interestingly, increased p53 expression in response to DNA damage did not result in elevated expression of p21. Our data demonstrated that the inflammation induced methylation of p21 promoter region. This methylation inhibited the expression of p21, the critical mediator of p53 function. The inhibition of p21 was reversed by treatment with the COX2 inhibitor Celecoxib and 5-aza 2′ deoxycytidine treatment. We believe that the loss of p15 and p16, combined with decreased expression of p21 is critical in dysregulation of cell cycle control. This explains a massive proliferation of epithelia in the forestomach of Tgfbr2^fspKO^ mice. Our data add novel mechanistic insight to the SCC development in addition to the finding of HGF as critical mediator [1] (Figure S7). Our data are supported by studies in which decreased levels or total loss of TGF-β signaling via defects of TGF-β receptors or Smads resulted in inflammation and uncontrolled proliferation of epithelial cells while promoting tumor development [26], [36], [47].

### Human ESCC

Our results in Tgfbr2^fspKO^ mice are clearly corroborated by human ESCC. Our studies showed significantly decreased expression of TβRII in FSP1+ stromal cells in human ESCC (Figure 6A). Interestingly, no alteration of TβRII was found in tumor epithelial cells compared to that of adjacent normal tissue [48], [49]. Similar to our mouse model data, elevated expression of inflammatory mediators such as COX2 and CCL2 as well as production of DNA damaging mediators 8-Oxo-dG and γ-H2AX were associated with the development of human ESCC (Figure 6C and 6D) [28], [48], [50]. Methylation of p21 gene promoter was also observed in 56% ESCC [28]. In addition, genetic deletion, loss of heterozygosity, and promoter methylation of p15 and p16 genes was associated with the development of human ESCC [30], [51], [52]. Co-deletion of p15 and p16 has also been found in human ESCC (Figure 6E) and [29], [49], [51]. Notably, approximately, 60% of human SCC including skin, head and neck, esophagus, bronchi, and uterine cervix are associated with the alterations in TGF-β signaling pathway molecules [53]. Indeed, stromal cell signaling has an impact on epithelial carcinogenesis and prediction of clinical outcome of cancer [54]. Furthermore, down-regulation of TβRII in tumor-associated stroma is correlated with poor prognosis in the clinic [55]. The squamous mucosal lining of mouse forestomach is similar to that of human esophagus at both histopathological and molecular levels [56]. Therefore, targeting inflammation may be a strategy to counteract the stromal-epithelial cross-talk in ESCC development.

## Methods

### Mice

Cre-Tgfbr2^flox/flox^ female and Cre+Tgfbr2^flox/wt^ male mice were kindly provided by Dr. Harold Moses, Vanderbilt Cancer center, Nashville, TN. Mice were bred to yield Tgfbr2^fspKO^ mice. Cre+Tgfbr2^flox/wt^ male mice were crossed with Rosa26 reporter female mice to validate the Cre specificity. All mice were housed at the National Cancer Institute (NCI) animal facility Animal studies were performed under NCI- IACUC approved protocol.

### Immunofluorescence and Immunohistochemistry

Forestomach samples were collected from Tgfbr2^fspKO^ and Tgfbr2^flox/flox^ mice, which then fixed, sectioned and stained using H&E. For immunofluorescence studies, the sections were incubated overnight at 4°C with primary antibodies directed against TβRII (1∶50, Santa Cruz), S100A4 (1∶100, Abcam), K14 (1∶100, Covance), F4/80 (1∶100, BD Transduction Laboratories), NOS2 (1∶100, BD Transduction Laboratories), Cox2 (1∶100, Cell Signaling Technology), p65 (1∶100, Cell Signaling Technology), γH2AX (1∶100, Trevigen), CD4 and IL17A (1∶100, Biolegend). Fluorescence-tagged secondary antibodies were used for visualization (anti-rabbit, 1∶1000, Invitrogen or; anti-mouse, Vector lab, respectively). Slides were examined using fluorescence microscopy (Olympus). For immunohistochemistry, slides were incubated with primary antibodies against CD45 (1∶100, BD Pharmigen), Psmad2 (1∶100, Cell Signaling Technology), and β-galactosidase (1∶100, Abcam). Signals were visualized using Vectastain (Vector Lab) and examined under a light microscope (Carl Zeiss). Quantitative data was measured by counting total number of cells expressing the marker out of all the cells in one field by Image J software. Three different fields were evaluated; percentage was calculated from total number of cells counted and averaged for three independent fields.

### Apoptosis Assay

Apoptosis was evaluated by TUNEL (Terminal deoxynucleotidyl transferase dUTP nick end labeling) method using “In Situ cell death detection kit, Fluorescein” (Roche Applied Science, Indianapolis, IN, USA). Briefly, tissue sections were pretreated with xylene and ethanol (100%, 95%, 90%, 80%, 70%), washed with 1× PBS, then treated with proteinase K (20 ug/ml in 10 mM Tris-HCL (pH 7.4) and 50 ul tunel mixture for 60 min. Data was acquired using fluorescence microscopy (Olympus). Quantitative data was obtained using Image J software by counting the number of cells expressing the marker out of total cells for each field. Three different fields were evaluated; the data was presented as percentage of stained cells in total cells and averaged for three independent fields.

### Flow Cytometry of Tumor Microenvironment

Single cell suspensions were made using fresh forestomach tissue from Tgfbr2^fspKO^ and Tgfbr2^flox/flox^ mice through incubation with Liberase TL (200 U/mL) (Roche Applied Science, Indianapolis, IN) at 37°C for 30 minutes. Forestomach tissue was then crushed and filtered through a 70 µm cell strainer. Cells were labeled with fluorescence-conjugated antibodies against CD45-PE, CD11b-FITC, Gr-1-APC, Ly6G-APC, Ly6C-PE, or 7-AAD (BD Pharmingen). Isotype-matched IgG was used as a control (BD Pharmingen). The flow data was acquired on BD FACS Calibur flow cytometer (BD Biosciences, San Jose, CA) and analyzed using FlowJO.

### Western Blotting

The forestomachs were dissected and treated with 0.05% trypsin overnight at 4°C. The forestomach tumor tissues from Tgfbr2^fspKO^ or equivalent normal tissues from Tgfbr2^flox/flox^ mice were separated by peeling them from the stromal and muscle layers. Protein was extracted, and then separated by gel electrophoresis. Membranes were incubated with primary antibodies against NOS2 (1∶100, BD Transduction Laboratories), γH2AX (1∶1000, Trevigen), HGF (1∶1000, Santa Cruz Biotechnology), Cox2, P-p65, P-p53, Cyclin D1, p21, p15, and p16 (all at 1∶1000, Cell Signaling Technology) or β-actin (1∶5000, Sigma), and horseradish peroxidase-conjugated secondary antibody (1∶5000, Biorad). The blots were developed using a SuperSignal West Pico Chemiluminescent substrate kit (Pierce). Images were scanned in a G: Box (Syngene).

### Pyrosequencing

DNA was isolated from epithelial layers of forestomach as described in “Western Blotting.” QIAGEN Genomic-tip 20/G (Qiagen, CA, USA) and modified with Epitech Bisulfite kit (Qiagen, CA, USA) were used in pyrosequencing. PCR templates for pyrosequencing analysis were amplified from 10 ng gDNA using Hotstart Taq Mastermix (Qiagen, CA, USA) and 5 pmol of each primer in a total reaction volume of 25 µl. In all, 1 µl of each PCR reaction was analysed on an Agilent 2100 Bioanalyzer (Santa Clara, CA) using a DNA 1000 kit. Pyrosequencing was carried out on 0.15–0.5 pmol of each PCR product using the PyroMark MD System (Qiagen, CA, USA) following the manufacturer's instructions with sequencing primers and assay parameters specific to each methylation site. Resulting pyrograms were analysed using the PyroMark MD 1.0 software in ‘AQ mode’. For each assay, duplicate pyrosequencing analysis was performed, and the average of these was taken to represent the identified percentage methylation of the methylated allele.

### Laser Captured Micro-Dissection

Laser capture microdissection of Tgfbr2^flox/flox^, Tgfbr2^fspKO^ and Celecoxib treated Tgfbr2^fspKO^ mouse tissue was performed using an Arcturus XT (Life Technologies, CA, USA). Frozen tissue sections on PEN membrane frame slides (Applied Biosystems) were H&E stained followed by dehydration using the standard protocol to improve visualization of the cells at the microscope. The epithelia and stroma were identified by morphology, captured using a low-power infrared laser pulse, and transferred onto a cap (Capsure™ Macro LCM Caps, Life Technologies). The DNA was extracted using a QIAamp DNA micro kit (Qiagen, CA, USA).

### 5-aza 2′ Deoxycytidine Treatments

Primary epithelial cells (1096, isolated from SCC of FSP-Cre/RII knock out mouse, and kept in low passages) were cultured in 6 well plate with seeding density 0.3×10^6^ per well for overnight in DME/F12 medium containing 10% FBS and 1× antibiotics (GIBCO, Life Technologies, CA, USA). DNA methyltransferase inhibitor, 5-aza 2′ deoxycytidine (Decitabine), was added to the culture at 5 uM concentration for 48 hours. Cells were harvested using 0.25% trypsin-EDTA (GIBCO, Life Technologies, CA, USA) and subjected to total RNA extraction by RNeasy mini kit (Qiagen, CA, USA) and protein extraction by standard method.

### RNA Isolation and Quantitative RT–PCR (qPCR)

Total RNA was isolated from laser captured micro-dissected tissue samples by Arcturus Picopure RNA isolation Kit (Applied Biosystems). Reverse transcription was performed using oligo dT primers and Superscript II (Invitrogen). The primers for qPCR were mCDKN1A (p21) F: 5′-ACAGGAGCAAAGTGTGCCGTTGT-3′; mCDKN1A (p21) R: 5′- GCTCAGACACCA GAGTGCAAGACA 3′; mGAPDH F: 5′-ATGACCACAGTCCATGCCATCACT-3′; mGAPDH R: 5′-TGTTGAAGTCGCAGGAGACAACCT-3′. PCR reactions were performed using fast real-time 7500 PCR system (Applied Biosystems). All samples were tested in triplicate. The comparative C_T_ method was used for quantification of gene expression. *Gapdh* was used as an endogenous reference. Statistical analysis was performed using SDS v2.1 software (Applied Biosystems) according to the manufacturer's instructions.

### Bio-Plex Protein Assays

Protein extraction was obtained from forestomach samples of Tgfbr2^fspKO^ and Tgfbr2^flox/flox^ mice, and analyzed for IFN-γ and TNF-α expression, as per manufacturer's instruction. Data was acquired and analyzed using Bio-Plex Manager version 4.0 software (Bio-Rad).

### Array-Based Comparative Genomic Hybridization (CGH)

Genomic DNA was isolated from primary tumor cells (1096) from *Tgfbr2^fspKO^* mice and primary normal epithelial cells isolated from forestomach epithelial cell layer with a QIAamp DNA Mini Kit according to manufacturer protocol (Qiagen, Valencia, CA). Array-CGH was performed using test DNA from laser captured epithelia and stroma, 1096 primary tumor cell culture, and reference DNA. DNA was labeled with Cy3 or Cy5 fluorescent dyes (Pharmacia, Piscataway, NJ) according to the BioPrime array CGH genomic labeling protocol (Invitrogen, Carlsbad, CA) and cleaned using Microcon YM-30 filters (Millipore, Billerica, MA). Hybridization was carried out using Mouse Genome CGH Microarray 4×44 K from Agilent Technologies (Santa Clara, CA) according to CGH Procedures for Genomic DNA Analysis (Agilent Technologies). Slides were hybridized for 20 hours, washed, and scanned with an Agilent microarray scanner. Data was analyzed using Feature Extraction® and CGH Analytics® software packages (Agilent Technologies). The array-based CGH data is available, GEO accession number: GSE42773.

### Genomic PCR

Genomic DNA from laser captured epithelia and stroma described above was used for genomic PCR using the 2× *Taq* master mix (Gene Script, NJ, USA), 50 ng genomic DNA and Exon 1 specific primers of mouse p15 and p16 genes. The primer sequences were p15: forward- 5′- GTT GGG CGG CAG CAG TGA C-3′ and reverse- 5′-CCT CCC GAA GCG GTT CAG-3′, p16: forward- 5′-ACT GGT CAC ACG ACT GGG CGA TTG -3′ and reverse- 5′-AAT CGG GGT ACG ACC GAA AGA G-3′. Actin: forward-5′-TCA TCA GGT AGT CAG TGA GGT CGC-3′ and reverse-5′-CAC CAC ACC TTC TAC AAT GAG CTG-3′. The PCR conditions included initial denaturation at 95°C for 5 min, denaturation at 95°C for 1 min, annealing at 60°C for 1 min and extension at 72°C for 1 min for 40 cycles and final extension at 72°C for 7 min. Agarose gel electrophoresis (2%) was used to detect the PCR products and data was recorded using G: Box (syngene).

### Mouse Line Re-Derivation

C57BL/6NCr wild type female mice were super-ovulated and crossed with the Fsp-Cre male mice. Four or eight cell embryos were transferred to pathogen free females. After birth, pups were examined for infection and genotypes using ELISA and PCR. Male mice with Cre+ Tgfbr2^flox/wt^ and female mice with Cre- Tgfbr2^flox/wt^ were identified and crossed to obtain pathogen free Tgfbr2^fspKO^ mice. The mice re-derivation was performed in the mice re-derivation core facility located at NCI-Frederick, MD. Mice were transferred and housed in Helicobacter free facility at NIH Bethesda, MD.

### Anti-Inflammation Treatment

Tgfbr2^flox/flox^ and Tgfbr2^fspKO^ mice were treated with diet pellets containing Celecoxib at 1500 ppm with (D04090202) or without (AIN 76A D1000) active compound, or *N*_ω_-Nitro-L-arginine methyl ester hydrochloride (L-NAME, Sigma) at a dose of 50 mg/kg/day. The pups received the Celecoxib starting the second week after birth, together with the nursing mother mouse in the same cage, due to early inflammation onset (Figure 1B). The treatment continued after weaning. The synergistic effect of L-NAME and Celecoxib was also examined. In addition, Mice housed in the Helicobacter free conditions were treated with Celecoxib to evaluate the cooperative effect in survival and phenotype. For gel diet treatment, after weaning on day 21, Tgfbr2^fspKO^ and Tgfbr2^flox/flox^ pups were fed with gel diet (AIN76A, Clear H_2_O). All mice were monitored daily and sacrificed with signs of poor health including small size, hunched body, slow movements, and weakness in comparison to healthy littermates.

### Analysis of Human Esophageal Squamous Cell Carcinoma (ESCC) and Breast Carcinoma

Human ESCC (n = 8) and adjacent normal FFPE tissue slides (n = 8) were previously described [57] (PMID: 19789312), and were stained to detect TβRII expression in FSP1+ stromal cells. p65, NOS2, and 8-Oxo-dG adducts were also examined for inflammation and DNA damage. The immunofluorescence staining procedures are as described above. Oncomine database (www.oncomine.com) was utilized to evaluate γ-H2AX expression, p15, and p16 loss in human ESCC, specifically mRNA expression datasets, Hu Esophagus (34 samples) and Su Esophagus 2 (106 samples), and DNA copy number datasets, Hu Esophagus 2 (blood vs LCM tumor sample) and Bass Esophagus (cell lines and tissue specimens). Dot plots of H2AX mRNA expression are presented as log2 median-centered intensity. The dataset (PMID: 19789312) from Oncomine was analyzed using GraphPad Prism 5.0 and two-tailed, paired t-test. Heat map of copy number loss of p15 and p16 in ESCC was obtained from the same dataset that measure DNA copy number on a SNP microarray platform. The expression of TβRII in tumor-associated stroma vs adjacent normal was also analyzed using Oncomine mRNA dataset Ma 4 Breast Carcinoma.

### Statistical Analysis

Data was analyzed using the Student t-test, and was expressed as mean ± SE. Differences were considered statistically significant at p<0.05. Mouse survival data was examined using SPSS 16 software, and is presented as Kaplan Meier curve. A log rank test was used to calculate statistical differences in survival and median survival of the different groups.

## Supporting Information

Figure S1 (A) H&E staining of Tgfbr2^fspKO^ forestomach tissue from various time points (E16, 1, 2, 3, 4, 5 weeks after birth) showed that hyperplasia began during week 3 and progressed to dysplasia by week 4 and invasive SCC by week 5. Scale bar: 50 µm (B) Immunofluorescence staining shows loss of TβRII expression in the stromal compartment of forestomach in Tgfbr2^fspKO^ mice (arrows). Original magnification: ×100. (C) FSP1-Cre mediated specific activity is demonstrated by positive β-galactosidase staining in the stromal compartment of forestomach tissue obtained by crossing FSP1-Cre and Rosa26 reporter mice (arrow). Scale bar: 20 µm. (D) Specific deletion of *Tgfbr2* is demonstrated by loss of nuclear p-SMAD2 staining in the stromal compartment (arrow) of the forestomach in Tgfbr2^fspKO^ mice compared to Tgfbr2^flox/flox^ mice. Scale bar: 20 µm. Str: Stroma; Epi: epithelia.
(TIF)

Figure S2 (A) Array-CGH of cancerous epithelial cell lines derived from Tgfbr2^fspKO^ mice indicating a loss of qC4 on chromosome 4 which include CDK inhibitors p15 and p16. (B) Laser captured microdissection (LCM) from forestomach tissue of Tgfbr2^flox/flox^ and *Tgfbr2^fspKO^* mice. The pictures show dissected epithelial and stromal samples before and after LCM. Three mice each for Tgfbr2^flox/flox^ and Tgfbr2^fspKO^ mice (left panel) were used for sample collection. (C) Array-CGH of laser dissected epithelial and stromal samples from the forestomach of Tgfbr2^fspKO^ mice showing a loss of p16 was found in epithelia but not stroma.
(TIF)

Figure S3 (A) Microscopy showing decreased proliferation of SCC tumor cells (1096 cell line) after 5-aza 2′ deoxycytidine treatment. This cell line was established from epithelial cell layer of forestomach tumor of Tgfbr2^fspKO^ mice. Shown is one of the two experiments performed. (B) Immunofluorescence microscopy of TUNEL assay in samples from Tgfbr2^fspKO^ mice showing increased apoptosis in the stromal area compared to that of epithelial. Quantitative data is listed below. Epi: epithelia; Str: stroma; Tu: tumor. red arrow: Tumor infiltrated stroma, white arrow: tumor cell.
(TIF)

Figure S4 (A) Significantly improved body weight (grams) of Tgfbr2^fspKO^ mice received Celecoxib treatment (n = 12), or under Helicobacter free housing condition (n = 9), compared to untreated Tgfbr2^fspKO^ mice (n = 11). No significant improvement was observed in gel diet treated Tgfbr2^fspKO^ (n = 5) or L-NAME treated Tgfbr2^fspKO^ (n = 12). (B) Line graph showing significantly decreased tumor burden in Tgfbr2^fspKO^ mice received Celecoxib treatment (n = 12), or under Helicobacter free housing condition (n = 9) at 5 weeks. Y axis is the stomach weight including the tumor tissues. Error bars represent SD. *P<0.05.
(TIF)

Figure S5 (A) No significant difference in the median survival of Tgfbr2^fspKO^ mice treated with L-NAME. Mouse number for treatment is indicated in the figure. (B) Quantitation of NO. in forestomach samples of Tgfbr2^flox/flox^ and Tgfbr2^fspKO^ mice. NO. was elevated in Tgfbr2^fspKO^ forestomach but significantly decreased after L-NAME treatment. Error bars represent SD. *P<0.05 and ***P<0.001. (C), H&E and Immunofluorescence staining of COX2 and p65 showing reduced hyperplasia/dysplasia in Celecoxib treated Tgfbr2^fspKO^ mice compared to untreated Tgfbr2^fspKO^ mice. Immunofluorescence staining indicated a decreased expression of COX2 and p65 in Tgfbr2^fspKO^ mice received Celecoxib treatment of in Helicobacter free condition. Scale bar: 50 µm.
(TIF)

Figure S6 (A) H&E staining of normal esophagus and advanced ESCC used in the studies for Figure 6. Scale bar: 50 µm. (B) Double immunofluorescence staining of FSP1 and macrophage marker F4/80 in Tgfbr2^fspKO^ mice (n = 3) compared to Tgfbr2^flox/flox^ mice (n = 3). There was no overlap of FSP1 and F4/80 in Tgfbr2^fspKO^ mice. Scale bar: 100 µm. Shown is one of the two experiments performed.
(TIF)

Figure S7 Time course studies of the HGF expression by Western blot, indicating that the HGF was not significantly elevated in tumor samples until mice were 5 week old.
(TIF)

Table S1 Detection of microbiome after Tgfbr2^fspKO^ mice rederivation (n = 7), rederivation showed absence of Helicobacter, Parvovirus and parasitic infections, and presence of Klebsiella pneumoniae infection.
(TIF)
