# Supplementary figures and images for: Inflammation-Mediated Genetic and Epigenetic Alterations Drive Cancer Development in the Neighboring Epithelium upon Stromal Abrogation of TGF-β Signaling
Source: PLoS Genet. 2013 Feb 7;9(2):e1003251. doi: 10.1371/journal.pgen.1003251 (PMC3567148; doi:10.1371/journal.pgen.1003251)

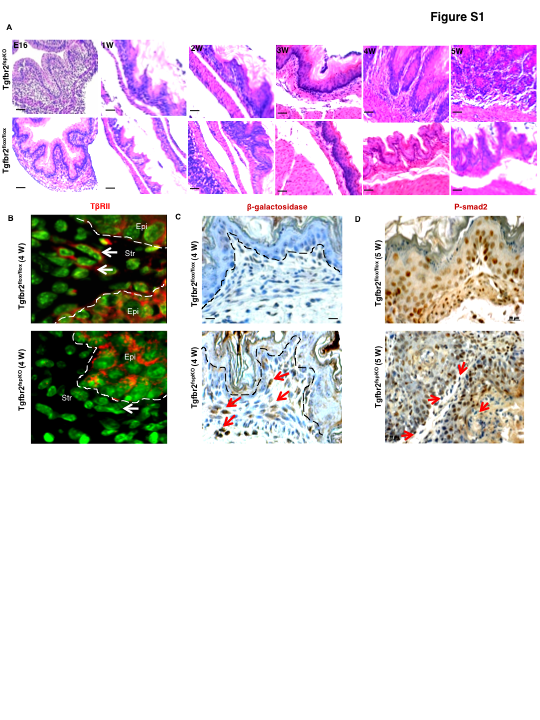

Supplement: Figure S1 — (A) H&E staining of Tgfbr2fspKO forestomach tissue from various time points (E16, 1, 2, 3, 4, 5 weeks after birth) showed that hyperplasia began during week 3 and progressed to dysplasia by week 4 and invasive SCC by week 5. Scale bar: 50 µm (B) Immunofluorescence staining shows loss of TβRII expression in the stromal compartment of forestomach in Tgfbr2fspKO mice (arrows). Original magnification: ×100. (C) FSP1-Cre mediated specific activity is demonstrated by positive β-galactosidase staining in the stromal compartment of forestomach tissue obtained by crossing FSP1-Cre and Rosa26 reporter mice (arrow). Scale bar: 20 µm. (D) Specific deletion of Tgfbr2 is demonstrated by loss of nuclear p-SMAD2 staining in the stromal compartment (arrow) of the forestomach in Tgfbr2fspKO mice compared to Tgfbr2flox/flox mice. Scale bar: 20 µm. Str: Stroma; Epi: epithelia. (TIF) [file pgen.1003251.s001.tif]

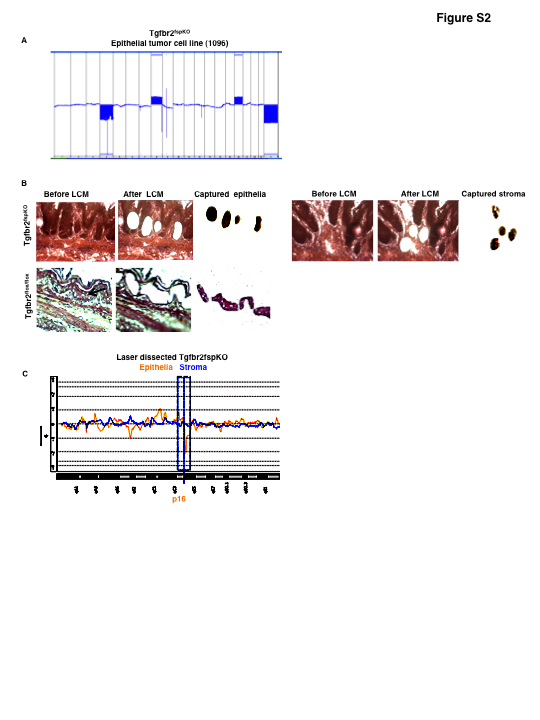

Supplement: Figure S2 — (A) Array-CGH of cancerous epithelial cell lines derived from Tgfbr2fspKO mice indicating a loss of qC4 on chromosome 4 which include CDK inhibitors p15 and p16. (B) Laser captured microdissection (LCM) from forestomach tissue of Tgfbr2flox/flox and Tgfbr2fspKO mice. The pictures show dissected epithelial and stromal samples before and after LCM. Three mice each for Tgfbr2flox/flox and Tgfbr2fspKO mice (left panel) were used for sample collection. (C) Array-CGH of laser dissected epithelial and stromal samples from the forestomach of Tgfbr2fspKO mice showing a loss of p16 was found in epithelia but not stroma. (TIF) [file pgen.1003251.s002.tif]

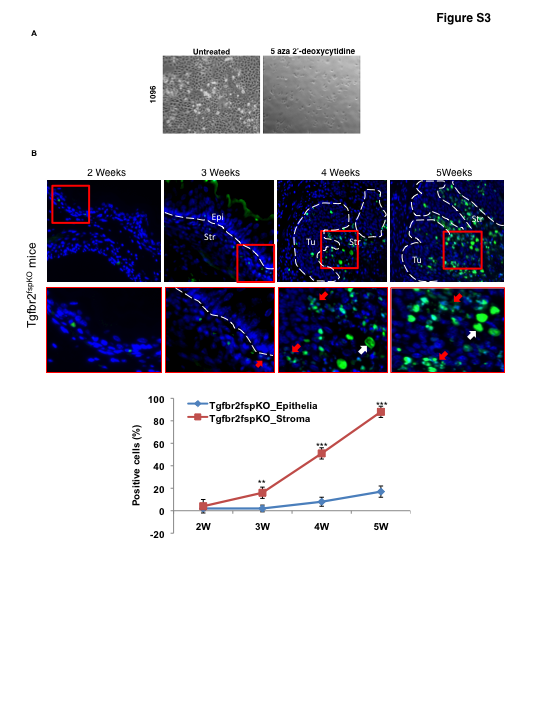

Supplement: Figure S3 — (A) Microscopy showing decreased proliferation of SCC tumor cells (1096 cell line) after 5-aza 2′ deoxycytidine treatment. This cell line was established from epithelial cell layer of forestomach tumor of Tgfbr2fspKO mice. Shown is one of the two experiments performed. (B) Immunofluorescence microscopy of TUNEL assay in samples from Tgfbr2fspKO mice showing increased apoptosis in the stromal area compared to that of epithelial. Quantitative data is listed below. Epi: epithelia; Str: stroma; Tu: tumor. red arrow: Tumor infiltrated stroma, white arrow: tumor cell. (TIF) [file pgen.1003251.s003.tif]

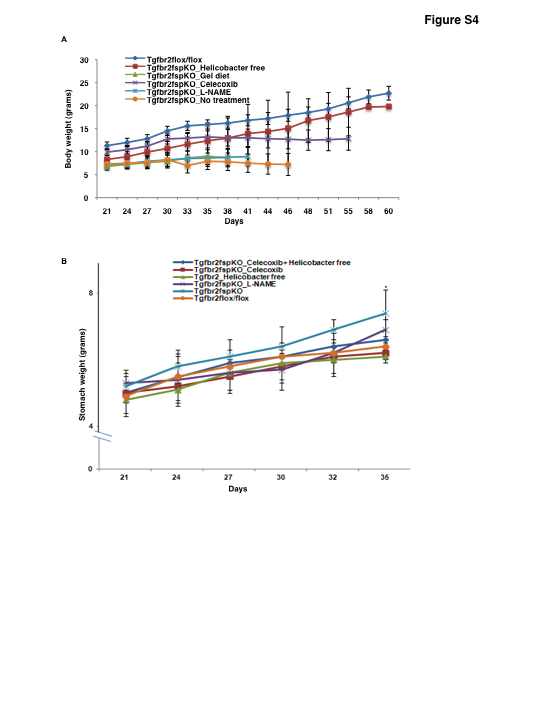

Supplement: Figure S4 — (A) Significantly improved body weight (grams) of Tgfbr2fspKO mice received Celecoxib treatment (n = 12), or under Helicobacter free housing condition (n = 9), compared to untreated Tgfbr2fspKO mice (n = 11). No significant improvement was observed in gel diet treated Tgfbr2fspKO (n = 5) or L-NAME treated Tgfbr2fspKO (n = 12). (B) Line graph showing significantly decreased tumor burden in Tgfbr2fspKO mice received Celecoxib treatment (n = 12), or under Helicobacter free housing condition (n = 9) at 5 weeks. Y axis is the stomach weight including the tumor tissues. Error bars represent SD. *P<0.05. (TIF) [file pgen.1003251.s004.tif]

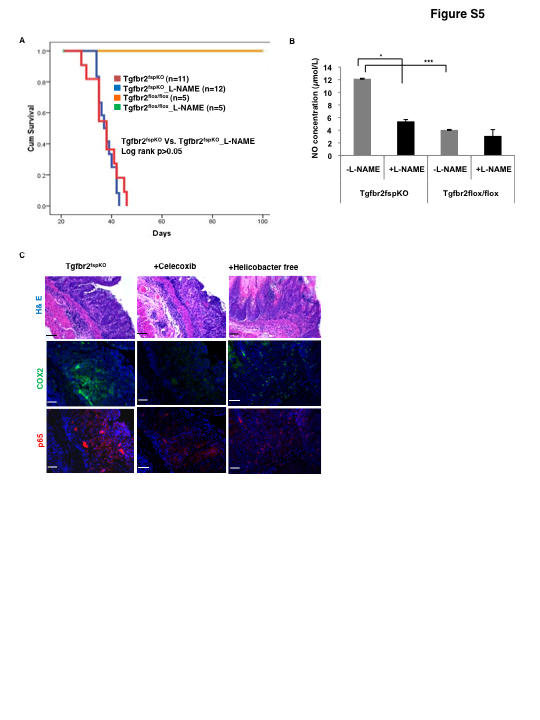

Supplement: Figure S5 — (A) No significant difference in the median survival of Tgfbr2fspKO mice treated with L-NAME. Mouse number for treatment is indicated in the figure. (B) Quantitation of NO. in forestomach samples of Tgfbr2flox/flox and Tgfbr2fspKO mice. NO. was elevated in Tgfbr2fspKO forestomach but significantly decreased after L-NAME treatment. Error bars represent SD. *P<0.05 and ***P<0.001. (C), H&E and Immunofluorescence staining of COX2 and p65 showing reduced hyperplasia/dysplasia in Celecoxib treated Tgfbr2fspKO mice compared to untreated Tgfbr2fspKO mice. Immunofluorescence staining indicated a decreased expression of COX2 and p65 in Tgfbr2fspKO mice received Celecoxib treatment of in Helicobacter free condition. Scale bar: 50 µm. (TIF) [file pgen.1003251.s005.tif]

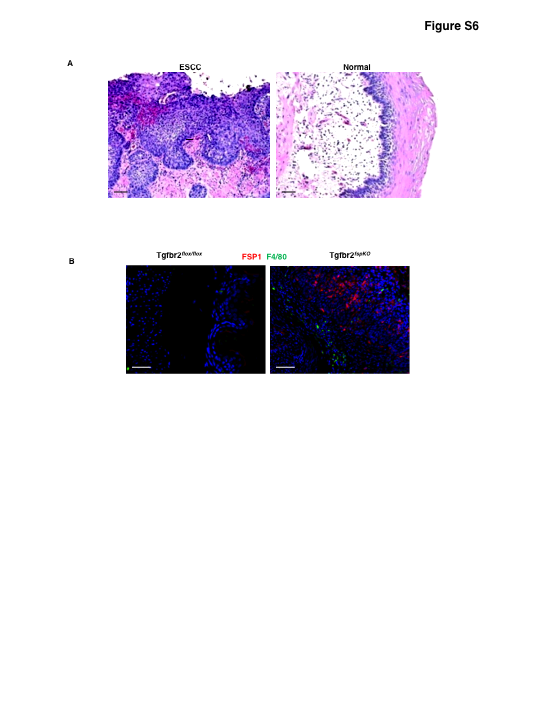

Supplement: Figure S6 — (A) H&E staining of normal esophagus and advanced ESCC used in the studies for Figure 6. Scale bar: 50 µm. (B) Double immunofluorescence staining of FSP1 and macrophage marker F4/80 in Tgfbr2fspKO mice (n = 3) compared to Tgfbr2flox/flox mice (n = 3). There was no overlap of FSP1 and F4/80 in Tgfbr2fspKO mice. Scale bar: 100 µm. Shown is one of the two experiments performed. (TIF) [file pgen.1003251.s006.tif]
